# Pediatric Pulmonology Year in Review 2024: Physiology

**DOI:** 10.1002/ppul.71269

**Published:** 2025-09-11

**Authors:** Heather Boas, Lucy Tan, Clement L. Ren

**Affiliations:** ^1^ Division of Pulmonary and Sleep Medicine, Children's Hospital of Philadelphia Philadelphia PA USA; ^2^ Department of Pediatrics University of Pennsylvania Perelman School of Medicine Philadelphia PA USA

**Keywords:** bronchopulmonary dysplasia, forced oscillometry, Multiple breath washout, pulmonary function testing, remote patient monitoring

## Abstract

Pulmonary physiology is central to pediatric pulmonology and pulmonary function testing, and in 2024 there were numerous studies published in *Pediatric Pulmonology* and other journals focused on this topic. This article will review some of the highlights over the past year.

## 1 Introduction

Pulmonary physiology and pulmonary function tests (PFTs) are fundamental elements of pediatric pulmonology. This was reflected in the numerous pediatric PFT papers published in *Pediatric Pulmonology* and other journals in 2024. This review will highlight some of the papers in this area.

### 1.1 Multiple Breath Washout

Multiple breath washout (MBW) measures functional residual capacity (FRC) and ventilation inhomogeneity (VI) [1]. MBW can be performed either by washing in a tracer gas, such as sulfur hexafluoride (SF_6_), and then washing the tracer gas out with ambient air or washing out the resident nitrogen (N_2_) in the lungs with 100% oxygen. The latter is faster to perform and does not require the use of a specialized gas. The lung clearance index (LCI) is the most commonly used measurement of VI derived from MBW, and for N_2_ MBW it represents the number of turnovers of the volume of gas in the lungs required to wash out resident N_2_ from the lungs down to 2.5% of the original concentration; a higher LCI indicates increased VI. Increased VI can be seen in conditions such as cystic fibrosis (CF), where airway inflammation and accumulation of airway mucus lead to impaired gas mixing [1]. Other MBW measurements include the convection dependent inhomogeneity component (S_cond_) and diffusion‐convection interaction‐dependent inhomogeneity component (S_acin_). S_cond_ is felt to reflect VI in the conducting airways, while S_acin_ reflects acinar inhomogeneity [1].

One challenge with obtaining and interpreting MBW results is that differences in devices, tracer gas, and software can affect the measurement [2]. There are 2 different software setups for infant MBW: WBreath and Spiroware. Oestrich, et al performed in vitro validation and characterization between these two setups using the same device [3]. Though not statistically significant, there were small differences in FRC measurements between the two setups. They conclude that data should not be used interchangeably between devices for infant MBW measurements.

Da Silva Sena, et al hypothesized that rhinovirus bronchiolitis and maternal asthma history may be associated with worse lung function outcomes at preschool age [4]. They compared N_2_ MBW results from preschool children (ages 3–5 years) with history of rhinovirus bronchiolitis (*n* = 39) to those from preschool children with rhinovirus negative bronchiolitis (*n* = 45; 90% were positive for respiratory syncytial virus). They found that the rhinovirus positive group had elevated S_cond_ at preschool age; there was also a significant association between rhinovirus and LCI at preschool age, which was strengthened with bronchiolitis severity, reflecting greater VI in the peripheral conducting airways in children with history of rhinovirus bronchiolitis.

Gambazza, et al investigated use of positive expiratory pressure (PEP) mask therapy (pressures 10‐15 cmH2O) as part of routine airway clearance in children and adolescents with CF [5]. In a multi‐center, randomized sham‐controlled crossover trial, 19 participants were randomized to either standard/sham or sham/standard sequence, with N_2_ MBW performed both before and after standard/sham therapy. S_acin_ was higher in the standard‐sham group, though not significantly so. Their results did not provide evidence for an immediate effect of PEP mask therapy on MBW outcomes in children with CF.

Urquhart, et al used N_2_ MBW to evaluate “real‐world” effectiveness of elexacaftor/tezacaftor/ivacaftor (ETI) in children with CF, as demonstrated by decrease in LCI [6]. They studied a cohort of 12 children ages 6–11 years who performed MBW both before and after initiation of ETI. They saw a trend towards improvement in LCI, with the largest decreases in individuals with higher pre‐ETI LCI. These results, while still demonstrating effectiveness of ETI, reflect that in the “real world” many children with normal pre‐ETI LCI may have less significant decreases in LCI as compared to phase 3 clinical trials.

Prenatal exposure to tobacco smoke (PTS) is associated with impaired airway function and increased risk of wheezing in infancy [7], but little is known about its effect on ventilation inhomogeneity (VI). To assess the effect of PTS on VI, Andrade, et al used SF_6_ MBW to measure LCI in 423 infants, 42 of whom were exposed to PTS [8]. Infants exposed to PTS had a significantly higher LCI compared to those without exposure, and their risk for wheezing was also significantly increased. These results expand our knowledge of the deleterious effects of PTS and provide further rationale for implementing smoking cessation initiatives in pregnant women.

LCI has been measured in infants with congenital diaphragmatic hernia (CDH) and is abnormal [9], but data in older children with CDH are lacking. Dirickx, et al obtained N_2_ MBW LCI on 29 children (median age 8.34 years) with a history of CDH and compared the results to chest computed tomography (CT) scores and spirometry [10]. LCI was abnormal in 50% of the children, whereas only 37.9% of children had an abnormal forced expiratory volume in 1 s (FEV1). There was a moderate correlation between LCI and both FEV1 and chest CT score. These results show that CDH affects multiple parts of respiratory function and suggest that measurement of LCI may be more sensitive than spirometry at detecting respiratory impairment in children with CDH.

As with any PFT, it is important to have normal reference values for LCI. The Global Lung Function Initiative recently collated LCI data from multiple PFT laboratories around the world to develop normal reference values for LCI and functional residual capacity across the age of 2–81 years [2]. A reference calculator is available at https://gli-calculator.ersnet.org/. Data from 1579 people were available, and although there were differences in MBW measurements obtained using different of devices and tracer gases, there was considerable overlap, leading the authors to conclude that a single reference equation was acceptable. However, measurements obtained using one device and tracer gas should not be used interchangeably with those from another.

### 1.2 Oscillometry

Oscillometry (OSC) (also known as forced oscillometry) measures respiratory system impedance (Z) by monitoring changes in pressure and flow in response to an oscillatory pressure wave introduced at the airway opening [11, 12]. Z incorporates the in‐phase and out‐of‐phase relationships between pressure and flow. Resistance (R) is composed of the forces associated with frictional losses in the airways and the lung parenchyma and is in‐phase with flow. Reactance (X) is composed of an inertive element (Xi), which represents the inertive forces of the respiratory system, and a capacitant element (Xc), which reflects the visco‐elastic properties of the lung; both components are out of phase with flow. At low frequencies, Xc predominates over Xi. Other measurements that can be derived from OSC include the resonant frequency (Fres) and the area under the reactance curve (AX). Fres is the frequency at which Xi=Xc and total X is zero. AX represents the area bounded by the X‐axis, X at 5 Hz, and Fres. Both Fres and AX are increased in the setting of obstructive airway disease [11]. At high frequencies, X is positive, and with lower frequencies X becomes more negative [11].

OSC can be performed using different input signals. Impulse oscillometry (IOS) uses an impulse pressure wave, whereas other devices use a pseudorandom noise signal [13]. Because OSC does not require a maximal forced expiratory maneuver, it may be easier to obtain measurements from young children compared to spirometry. Most, but not all studies in young children have demonstrated a higher success rate in obtaining acceptable OSC data compared to spirometry [14, 15, 16, 17]. OSC may also be superior to spirometry in assessing small airways function [18].

Oscillator mechanics are usually described in mathematical terms where Z is expressed as a complex number consisting of a real component (R) and an imaginary component (X) [19]. This framework can sometimes be challenging to appreciate. In a letter to the editor, Vamos and Allen present a more intuitive approach to oscillation mechanics using an analogy to musical and stringed instruments [20]. For example, in musical instruments lower resonant (or natural) frequency correlates with a larger string or instrument size; similarly decreasing resonant frequency of the respiratory system is associated with growth. Such simple musical analogies can help describe oscillation mechanics without use of complex mathematical equations.

Measurement of bronchodilator responsiveness (BDR), a feature of asthma, is commonly performed in the assessment of children with possible asthma, but many preschool aged (3‐5 years) children have difficult performing spirometry‐based BDR. Meoli, et al investigated using IOS and spirometry to assess this in a cohort of preschool children [21]. Of the 36 patients studied, 7 had positive BDR as determined by spirometry, while 4 had a positive BDR by IOS using European Respiratory Society (ERS) criteria [22]. Notably, no patient had a positive BDR with both PFTs. However, rather than using ERS criteria for IOS BDR, they found the best sensitivity and specificity to detect an increase in FEV1 of 12% was a decrease in R5 of 25.7% or an increase in X5 of 25.7%. Their results support use of OSC in the diagnosis of asthma and BDR, particularly in the preschool population.

To assess the impact of COVID‐19 infection on long‐term lung function, Mercan, et al assessed respiratory function in 65 children ages 3–15 years by performing IOS within 3–6 months following confirmed infection with COVID‐19, matched with 57 healthy controls [23]. In the COVID‐19 group, R was higher, while X was lower as compared to healthy controls. These findings suggest that even mild COVID‐19 infections can affect respiratory function in both very young children as well as adolescents.

To assess the utility of field‐based PFTs, Lee, et al sent research respiratory therapists to the homes of 76 school‐aged children with bronchopulmonary dysplasia (BPD) and performed oscillometry and spirometry on 2 separate visits [24]. They were able to successfully obtain acceptable data in a higher proportion of home‐based oscillometry tests than spirometry (95% vs 71% for the first visit and 98% vs 77% for the second visit). Moderate statistical correlations were observed between spirometry and oscillometry measurements, but oscillometry was significantly less likely than spirometry to detect respiratory abnormalities, Therefore, while oscillometry performed outside the PFT lab may be more technically feasible in school‐aged children with BPD, spirometry was more likely to detect abnormalities, highlighting the need for further investigation to understand the complementary roles of each in monitoring and characterizing pediatric lung disease.

### 1.3 Infant Pulmonary Function Testing

In 2024, infant PFTs were used in many studies published in *Pediatric Pulmonology*. Infant PFT techniques used included thoraco‐abdominal compression (RTC) at FRC or with raised volumes, which replicates adult‐type spirometry in infants, passive expiratory mechanics measured using the single breath occlusion technique (SBOT), and tidal breathing measurements [25, 26, 27].

Little is known about lung function in infants with primary ciliary dyskinesia (PCD). Koucký, et al performed a wide range of infant PFTs (MBW, body plethysmography, SBOT, and RTC at FRC) in cohort of 15 infants with PCD and 16 normal control infants at a median age of 39.5 weeks [28]. The maximal flow at functional residual capacity was significantly lower in infants with PCD compared to normal controls, while there were no significant differences between any of the other measurements. These results demonstrate that pulmonary function abnormalities can be detected in infants with PCD and highlight the importance of early diagnosis and treatment of this disease.

The peak inspiratory pressure (PIP) delivered by mechanical ventilation is dissipated in overcoming airway resistance (Rrs) and the visco‐elastic properties (compliance) of the respiratory system (Crs). The pressure needed to overcome Crs can be determined by measuring the plateau pressure (Pplat), which in turn is obtained by pausing the ventilator at end inspiration [29]. During the pause, flow falls to zero, and no pressure is required to overcome Rrs. Because Pplat can be more difficult to measure in young children, Buratti, et al sought to determine if PIP can accurately estimate Pplat in children with respiratory failure due to severe respiratory viral infection (SRVI). They retrospectively reviewed the records of 37 children (median age 3 months) who were receiving mechanical ventilation due to SRVI. They found that PIP overestimated Pplat by a mean difference of 7.3 cm H_2_O. Their results indicate that PIP cannot substitute for Pplat and that measurement of pressure under static conditions is required to accurately determine the contribution from Rrs and Crs.

Impaired diaphragm function commonly occurs in preterm infants, but the effect of an external inspiratory resistance load on diaphragm function in preterm infants has not been reported previously. Dassios, et al measured transdiaphragmatic pressure and airflow in 17 term infants and 23 preterm infants (6 with BPD and 17 without). The median pre‐resistance diaphragmatic pressure‐time index (PTIdi) was higher in preterm infants compared to term infants, and it was higher in preterm infants with BPD compared to those without BPD, indicating baseline impaired diaphragm function that is worse in infants with more severe lung disease. After application of an inspiratory flow resistance, PTIdi was again higher in preterm infants compared to term infants and higher in BPD compared to no BPD. These results show that premature birth is associated with impaired diaphragm function that worsens if an inspiratory resistance load, e.g. viral upper respiratory infection, is applied.

Physiologic findings in BPD include increased airway resistance and reduced lung compliance [30, 31], both of which can increase the work of breathing (WOB) for infants with BPD. Measuring WOB to help guide management of noninvasive respiratory support (NRS) has been studied in younger infants with BPD, but similar data in older infants are lacking. Dudoignon, et al assessed WOB at a mean age of 165 days in 8 infants with severe BPD, all of whom were receiving NRS, by measuring esophageal pressure (Pes) and gastric pressure (Pgas) to calculate transdiaphragmatic pressure. In 4 out of the 8 infants, changes in NRS were made based on the physiologic assessment, but because of the long time it took to wean infants off NRS, no conclusions about the impact of these assessments on weaning time could be determined. The results of this small study suggest that measuring WOB may be useful in managing infants with severe BPD requiring NRS, but larger, prospective studies are needed.

Infants with CDH can be managed with conventional mechanical ventilation (CMV) or high frequency oscillatory ventilation (HFOV), and it is unclear which is the best initial ventilation modality. Kimura, et al performed a retrospective analysis of 23 infants with CDH in whom static respiratory mechanics were measured. In all infants, HFOV was the initial ventilation modality. Infants who were converted to CMV had a higher Rrs compared to those who remained on HFOV, suggesting that physiologic measurements may have a role in selection of ventilation mode in infants with CDH. However, larger prospective studies are needed to fully assess the role of physiologic measurements in the management of mechanical ventilation in infants with CDH.

Lung agenesis (LA) is a rare condition, and little is known about early life lung function in infants with LA. Nobile, et al performed tidal breathing analysis and MBW in an infant girl with LA [32]. Tidal volumes increased and respiratory rate decreased from 2 weeks of age to 15 months. The ratio of time to peak expiratory flow over total expiratory time (Tpef/Te) decreased slightly, which may indicate flow obstruction as lung growth occurred in the remaining lung [33, 34, 35]. LCI was measured only once, but it was elevated at 9.91. These results show that compensatory growth occurs in LA, but lung function is not entirely normal at 15 months.

Dysanapsis refers to disproportionate growth of the airways relative to the lung parenchyma, and it has been noted to be increased in obese children with asthma [36, 37]. The goal of a study done by Sanchez‐Solis, et al was to assess the relationship between BMI and weight gain in infancy and infant PFTs using the raised volume RTC technique [38]. Both a higher BMI and higher weight gain were associated with lower forced expiratory volume in 0.5 s over forced vital capacity ratio, suggestive of dysanapsis. Their results suggest that the impact of obesity on lung development occurs early in life, and that interventions to prevent obesity‐related pulmonary complications need to be initiated in infancy.

Polyunsaturated fatty acids (PUFA) have anti‐inflammatory properties, and in a recent randomized clinical trial (RCT) preterm infants who received PUFA supplementation had less inflammation and required fewer days of respiratory support compared to the placebo group. Wendel, et al measured lung function at 3 months chronologic age in participants of the RCT using tidal breathing analysis [39]. They found no significant difference in Tpef/Te or tidal volume in PUFA treated infants compared to placebo. However, BPD was associated with a lower Tpef/Te and higher linear growth was associated with larger tidal volumes, highlighting the impact of BPD on respiratory function and the importance of growth and nutrition in preterm infants.

### 1.4 Spirometry and Reference Equations

#### 1.4.1 Race Neutral Reference Equations

Interpretation of PFT results requires having normal reference values. Race was historically considered an important variable to account for in establishing normal reference values, but more recently this concept has been challenged and reconsidered [40, 41]. Although genetic ancestry contributes to differences in PFT values in different populations [42, 43, 44], race and ethnicity are not a substitute for genetic ancestry, and other factors not captured in genetic ancestry, such as environmental exposures, can also affect normal lung function. In recognition of these concerns, the American Thoracic Society (ATS) published a statement in 2023 recommending against the use of race‐specific PFT reference equations [45]. Instead, the Global Lung Function Initiative (GLI) average equation (GLI Global) [46], a weighted average of the original race‐specific GLI reference equations, should be used. It is important to note that the use of the term “race neutral” does not refer to the equations themselves, which are a composite of race‐specific equations; rather, “race neutral” refers to the fact that race is not incorporated into selection of the reference equation.

In 2024, there were several studies of the impact of changing from race‐specific equations to GLI Global. Forno, et al analyzed spirometry data from 24,630 children ages 6–21 years at 2 separate pediatric PFT laboratories, comparing z‐scores and percent predicted values using race‐specific GLI equations [47] to GLI Global equations [48]. They found that using GLI Global equations Black children were more likely to have a decrease in FEV1 and forced vital capacity (FVC) *z*‐scores and an increase in FEV1/FVC z‐scores. Similar findings were seen in other races (including Asian and mixed), while White children were more likely to have increased FEV1 and FVC *z*‐scores, with decreased FEV1/FVC *z*‐score. These patterns were also true regarding percent predicted values. Black children were also more likely to have a change in spirometry pattern from normal to restrictive, while more White children changed to a normal pattern. The authors speculate that switching to GLI Global is likely to affect the management of children with chronic lung disease and that future studies on the impact of these changes are needed.

To investigate changes in reference equations on people with CF (PwCF), Rosenfeld, et al analyzed data from the CF Foundation Patient Registry [49]. They examined spirometry results from a cohort of 24,279 PwCF ages ≥ 6 years and compared FEV1 percent predicted (FEV1pp) values using GLI 2012 and GLI Global. Similar to the findings of Forno, et al, they found a general increase in FEV1pp in White PwCF, with a general decrease in FEV1pp in Black PwCF and those of other races. The change in FEV1pp has the potential to affect referral for lung transplantation and clinical trial eligibility. Notably, greater impacts on FEV1pp were seen in those with higher FEV1. Since children with CF tend to have higher FEV1pp, the change to GLI Global is likely to impact more children than adults.

Together, these two studies highlight the clinical implications of the recommended change to GLI Global equations. They show that respiratory disease and severity has likely been underestimated amongst Black and minority races and that future work is needed to understand the impact these changes will have on more equitable diagnosis and treatment of children with lung disease.

#### 1.4.2 Home Spirometry

The COVID‐19 pandemic led to an increased use of telehealth and remote patient monitoring, including home spirometry [50, 51, 52]. Numerous studies of home spirometry were reported in *Pediatric Pulmonology* in 2024.

To evaluate the impact of telehealth and home spirometry on CF disease progression and care, Medbo, et al performed a prospective multicenter study in Sweden with 59 participants over a mean study period of 6.8 months [53]. There was no significant difference between the pre‐pandemic and intervention periods in mean annual change in FEV1pp on hospital spirometry, mean annual changes in LCI, major shifts in overall incidence of airway pathogens, or use of antibiotics. Although home spirometry measurements were consistently higher and more variable than those obtained in the hospital, the overall longitudinal trends in FEV1pp were similar between home and hospital‐based spirometry. Questionnaire responses revealed that 39% of participants and 61% of caregivers felt more comfortable with the availability of home spirometry and reported various benefits, including reduced travel time and less disruption to work and school. The study demonstrated that incorporating both telehealth and home spirometry alongside in‐person visits yields clinical outcomes comparable to standard in‐person care with added benefits of flexibility and personalization.

Bouteleux, et al. conducted a retrospective study to assess the utility of home spirometry in detecting early FEV1 recovery patterns following intravenous (IV) antibiotic treatment for pulmonary exacerbations (PEx) in 65 children with CF using telemonitoring devices [54]. The analysis included symptom reports and home spirometry data collected from 45 days before to 60 days after 346 PEx requiring IV antibiotics. A significant decrease in home FEV1 was observed 8 days before initiation of antibiotics. The findings support the use of home spirometry to monitor trends in lung function before and after CF PEx.

There are limited data regarding the use of home spirometry among pediatric asthma populations. Burbank, et al performed a prospective cohort study to determine if unsupervised home‐based spirometry could detect clinically significant declines in FEV1 that corresponded to early loss of asthma control [55]. Over a 6‐month study period, a cohort of 42 adolescents performed unsupervised spirometry and symptom surveys twice daily. There was no significant change in home‐based FEV1 measurements during mild loss‐of‐control events. These findings suggest there may be limited utility of home spirometry in detecting mild loss of asthma control in adolescent patients, and further studies are needed to evaluate its utility in identifying more severe exacerbations.

#### 1.4.3 Other Spirometry Papers

Measuring BDR is commonly performed during PFTs, but there have been differing definitions of BDR in children. The 2005 ATS/ERS guidelines define a positive BDR as an increase in pre‐bronchodilator FEV1 or FVC of ≥ 12% of the baseline value and at least ≥ 200 mL increase (12% of the baseline) [56]. The 2021 ERS/ATS guidelines, on the other hand, recommended changing the definition of a positive BDR to an increase in pre‐bronchodilator FEV1 or FVC of ≥ 10% of the predicted value [57]. To compare BDR classification according to both sets of guidelines, Beydon and Rosenfeld analyzed 1365 pre‐and post‐bronchodilator spirometry tests among 1224 children with asthma or chronic cough in Paris, France [58]. Implementation of the 2021 BDR criteria resulted in a small yet significant increase in BDR‐positive tests relative to the 2005 guidelines, particularly in those with high baseline FEV1 measurements and minimal airflow obstruction. A small subset of children with low baseline FEV1 and significant obstruction were reclassified from BDR‐positive to BDR‐negative under the 2021 definition, as a 12% improvement represents a smaller absolute change when compared to 10% of predicted FEV1. Therefore, adoption of the 2021 BDR criteria may improve diagnostic sensitivity of asthma in children with preserved lung function and prompt reevaluation of treatment strategies in children with more pronounced airflow obstruction.

The FEV1/FVC ratio typically follows the “Shepherd's Hook” pattern, decreasing during childhood, briefly increasing in early adolescence, and then gradually declining throughout life [59, 60]. To determine if this pattern is also observed in children with asthma, Ahmed, et al performed a cross‐sectional retrospective analysis on spirometry from 1,793 children with persistent asthma [61]. Asthmatic children followed a trajectory to that of non‐asthmatic children but with lower ratios beginning at age 5 that persisted across all ages. From ages 5 to 11, the decline in FEV1/FVC was proportionally less in asthmatic children. After bronchodilator administration, the curvilinear trend persisted but appeared blunted. A comparable curvilinear pattern was also seen in obese children with asthma, though their ratios were persistently lower than those of healthy children. These findings demonstrate that although FEV1/FVC ratios in children with persistent asthma follow the characteristic ‘Shepherd's Hook’ developmental pattern, they remain consistently lower, indicating greater airway obstruction and a potential risk for lifelong asthma or progression to chronic obstructive pulmonary disease.

The isolated low FEV1 spirometry pattern (ILFSP), defined as an FEV1 below the lower limit of normal with preserved FVC and FEV1/FVC [62], has been studied in adults, but not in children. Wyatt et al completed a single‐center retrospective analysis of 29,979 spirometry tests from 8128 children with suspected or diagnosed respiratory conditions to evaluate the prevalence, stability, and clinical significance of ILFSP [63]. Most of the PFTs showed a normal spirometry pattern (61%), but 2% demonstrated ILFSP. Spirometry quality in a subset of 50 tests with ILFSP was deemed satisfactory, suggesting this pattern was not due to a problem with technique. A primary diagnosis of CF was the baseline characteristic most strongly associated with ILFSP with odds ratio 8.37 (*p* < 0.001). Compared to children with CF and normal spirometry, those with ILFSP exhibited markers of greater disease severity. The findings from this study highlight ILFSP as a distinct and relatively common pattern in certain pediatric populations, such as those with CF. The strong association between ILFSP and increased disease severity in CF challenges the prevailing view of this pattern as normal and underscores the need for further investigation and clinical consideration. In correspondence related to Wyatt, et al, Abdesslem and Ben Saad reported a similar ILFSP prevalence of 2.65% in Tunisian children [64]. These findings highlight the recurring presence of this spirometry pattern across different geographic regions and suggest it may represent a recognizable pattern among pediatric patients with suspected or diagnosed respiratory conditions.

## 2 Summary

Pulmonary physiology continues to occupy a central role in pediatric pulmonology, as evidenced by the many insightful and informative papers published in 2024 in *Pediatric Pulmonology*. We anticipate a similar pattern in 2025.

## Author Contributions

**Heather Boas:** writing – original draft, methodology, validation, writing – review and editing, formal analysis. **Lucy Tan:** writing – original draft, methodology, validation, writing – review and editing, formal analysis. **Clement L Ren:** conceptualization, writing – original draft, methodology, validation, writing – review and editing, formal analysis, project administration, resources, supervision, data curation.

## Conflicts of Interest

The authors declare no conflicts of interest.

## Data Availability

Data sharing not applicable to this article as no datasets were generated or analyzed during the current study.

## References

[1] P. D. Robinson , M. D. Goldman , and P. M. Gustafsson , “Inert Gas Washout: Theoretical Background and Clinical Utility in Respiratory Disease,” Respiration 78, no. 3 (2009): 339–355. 19521061 10.1159/000225373

[2] K. A. Ramsey , S. Stanojevic , L. Chavez , et al., “Global Lung Function Initiative Reference Values for Multiple Breath Washout Indices,” European Respiratory Journal 64, no. 6 (2024): 2400524. 39326920 10.1183/13993003.00524-2024

[3] M.‐A. Oestreich , R. Hänni , F. Wyler , et al, “Comparison of Functional Residual Capacity Between Updated Infant SF6 Multiple‐Breath Washout Setups,” Pediatric Pulmonology 59, no. 2 (2024): 518–520. V. 38010843 10.1002/ppul.26770

[4] C. R. Da Silva Sena , M. Morten , J. Meredith , et al., “Rhinovirus Bronchiolitis, Maternal Asthma, and the Development of Asthma and Lung Function Impairments,” Pediatric Pulmonology 56, no. 2 (2021): 362–370. 33179407 10.1002/ppul.25165

[5] S. Gambazza , A. Mariani , R. Guarise , et al., “Short‐Term Effects of Positive Expiratory Pressure Mask on Ventilation Inhomogeneity in Children With Cystic Fibrosis: A Randomized, Sham‐Controlled Crossover Study,” Pediatric Pulmonology 59, no. 5 (2024): 1354–1363. 38362833 10.1002/ppul.26915

[6] D. S. Urquhart , H. Dowle , K. Moffat , J. Forster , S. Cunningham , and K. A. Macleod , “Lung Clearance Index (LCI2.5) Changes After Initiation of Elexacaftor/Tezacaftor/Ivacaftor in Children With Cystic Fibrosis Aged Between 6 and 11 Years: The “Real‐World” Differs From Trial Data,” Pediatric Pulmonology 59, no. 5 (2024): 1449–1453. 38415920 10.1002/ppul.26938

[7] C. T. McEvoy and E. R. Spindel , “Pulmonary Effects of Maternal Smoking on the Fetus and Child: Effects on Lung Development, Respiratory Morbidities, and Life Long Lung Health,” Paediatric Respiratory Reviews 21 (2017): 27–33. 27639458 10.1016/j.prrv.2016.08.005 PMC5303131

[8] E. De Queiroz Andrade , C. R. D. S. Sena , P. de Gouveia Belinelo , et al., “In Utero Smoking Exposure Induces Changes to Lung Clearance Index and Modifies Risk of Wheeze in Infants,” Pediatric Pulmonology 59, no. 6 (2024): 1686–1694. 38501326 10.1002/ppul.26975

[9] A. Dotta , S. Palamides , A. Braguglia , et al., “Lung Volumes and Distribution of Ventilation in Survivors to Congenital Diaphragmatic Hernia (CDH) During Infancy,” Pediatric Pulmonology 42, no. 7 (2007): 600–604. 17526007 10.1002/ppul.20609

[10] M. Dirickx , F. Vermeulen , M. Boon , A. Debeer , and M. Proesmans , “Multiple Breath Washout Measurements in School Aged Patients With Congenital Diaphragmatic Hernia,” Pediatric Pulmonology 59, no. 5 (2024): 1493–1497. 38289110 10.1002/ppul.26888

[11] M. D. Goldman , “Clinical Application of Forced Oscillation,” Pulmonary Pharmacology & Therapeutics 14, no. 5 (2001): 341–350. 11603948 10.1006/pupt.2001.0310

[12] H. D. Komarow , I. A. Myles , A. Uzzaman , and D. D. Metcalfe , “Impulse Oscillometry in the Evaluation of Diseases of the Airways in Children,” Annals of Allergy, Asthma & Immunology 106, no. 3 (2011): 191–199. 10.1016/j.anai.2010.11.011 PMC3401927 21354020

[13] N. Beydon , S. D. Davis , E. Lombardi , et al., “An Official American Thoracic Society/European Respiratory Society Statement: Pulmonary Function Testing in Preschool Children,” American Journal of Respiratory and Critical Care Medicine 175, no. 12 (2007): 1304–1345. 17545458 10.1164/rccm.200605-642ST

[14] T. W. Guilbert , W. J. Morgan , R. S. Zeiger , et al., “Long‐Term Inhaled Corticosteroids in Preschool Children at High Risk for Asthma,” New England Journal of Medicine 354, no. 19 (2006): 1985–1997. 16687711 10.1056/NEJMoa051378

[15] T. W. Guilbert , A. M. Singh , Z. Danov , et al., “Decreased Lung Function After Preschool Wheezing Rhinovirus Illnesses in Children at Risk to Develop Asthma,” Journal of Allergy and Clinical Immunology 128, no. 3 (2011): 532–538.e10. 21878241 10.1016/j.jaci.2011.06.037 PMC3233203

[16] M. Kattan , L. B. Bacharier , G. T. O'Connor , et al., “Spirometry and Impulse Oscillometry in Preschool Children: Acceptability and Relationship to Maternal Smoking in Pregnancy,” Journal of Allergy and Clinical Immunology: In Practice 6, no. 5 (2018): 1596–1603.e6. 29449165 10.1016/j.jaip.2017.12.028 PMC6089669

[17] G. S. Kerby , M. Rosenfeld , C. L. Ren , et al., “Lung Function Distinguishes Preschool Children With CF From Healthy Controls in a Multi‐Center Setting,” Pediatric Pulmonology 47, no. 6 (2012): 597–605. 22081559 10.1002/ppul.21589

[18] M. van den Berge and H. A. M. Kerstjens , “Finally More Direct Evidence That Impulse Oscillometry Measures Small Airway Disease,” American Journal of Respiratory and Critical Care Medicine 200, no. 8 (2019): 951–952. 31188643 10.1164/rccm.201905-1037ED PMC6794106

[19] H. Smith , R. Reinhold , and M. Goldman , “Forced Oscillation Technique and Impulse Oscillometry,” European Respiratory Journal 31 (2005): 72–105.

[20] L. Vamos and J. Allen , “The Use of Stringed Instruments as An Analogy to Explain Oscillation Mechanics of the Developing Normal and Diseased Respiratory System,” Pediatric Pulmonology 59, no. 4 (2024): 1110–1113. 38226848 10.1002/ppul.26846 PMC10978273

[21] A. Meoli , J. Trischler , M. Hutter , et al., “Impulse Oscillometry Bronchodilator Response in Preschool Children,” Pediatric Pulmonology 59, no. 5 (2024): 1321–1329. 38353391 10.1002/ppul.26909

[22] G. G. King , J. Bates , and K. I. Berger , et al., “Technical Standards for Respiratory Oscillometry,” European Respiratory Journal 55, no. 2 (2020): 1900753. 31772002 10.1183/13993003.00753-2019

[23] A. Mercan , S. A. Beşe , Z. G. Köksal , S. S. Kara , P. Uysal , and D. Erge , “Impulse Oscillometry Assessment of Respiratory Function in Pediatric Patients With a History of COVID‐19,” Pediatric Pulmonology 59, no. 5 (2024): 1394–1401. 38390766 10.1002/ppul.26926

[24] J. X. Lee , M. Ryan , L. Mukharesh , et al., “Comparison of Home‐Based Spirometry and Oscillometry Measurements in School‐Age Children With Bronchopulmonary Dysplasia,” Pediatric Pulmonology 59, no. 10 (2024): 2589–2596. 38804690 10.1002/ppul.27072

[25] D. J. Weiner , J. L. Allen , and H. B. Panitch , “Infant Pulmonary Function Testing,” Current Opinion in Pediatrics 15, no. 3 (2003): 316–322. 12806264 10.1097/00008480-200306000-00016

[26] A. Feher , R. Castile , J. Kisling , et al., “Flow Limitation in Normal Infants: A New Method for Forced Expiratory Maneuvers From Raised Lung Volumes,” Journal of Applied Physiology 80, no. 6 (1996): 2019–2025. 8806909 10.1152/jappl.1996.80.6.2019

[27] J. Palmer , J. Allen , and O. Mayer , “Tidal Breathing Analysis,” NeoReviews 5, no. 5 (2004): e186–e193.

[28] V. Koucký , V. Martinů , and M. Koucký , “Impaired Lung Function in Infants With Primary Ciliary Dyskinesia: A Pilot Czech Study,” Pediatric Pulmonology 59, no. 4 (2024): 1124–1127. 38206044 10.1002/ppul.26863

[29] R. C. Bone , “Compliance and Dynamic Characteristics Curves in Acute Respirator Failure,” Critical Care Medicine 4, no. 4 (1976): 173–179. 939112 10.1097/00003246-197607000-00001

[30] E. Baraldi , M. Filippone , D. Trevisanuto , V. Zanardo , and F. Zacchello , “Pulmonary Function Until Two Years of Life in Infants With Bronchopulmonary Dysplasia,” American Journal of Respiratory and Critical Care Medicine 155 (1997): 149–155. 9001304 10.1164/ajrccm.155.1.9001304

[31] A. Bhandari and H. B. Panitch , “Pulmonary Outcomes in Bronchopulmonary Dysplasia,” Seminars in Perinatology 30, no. 4 (2006): 219–226. 16860162 10.1053/j.semperi.2006.05.009

[32] S. Nobile , P. Catalano , A. Perri , et al., “Lung Agenesis in a Preterm Infant: Lung Function Test Findings in the First 2 Years of Life,” Pediatric Pulmonology 59, no. 10 (2024): 2644–2646. 38751017 10.1002/ppul.27042

[33] P. Latzin , S. Roth , C. Thamrin , et al., “Lung Volume, Breathing Pattern and Ventilation Inhomogeneity in Preterm and Term Infants,” PLoS ONE 4, no. 2 (2009): e4635. 19247491 10.1371/journal.pone.0004635 PMC2645689

[34] F. D. Martinez , W. J. Morgan , A. L. Wright , C. J. Holberg , and L. M. Taussig , “Diminished Lung Function as a Predisposing Factor for Wheezing Respiratory Illness in Infants,” New England Journal of Medicine (1988): 319. 10.1056/NEJM198810273191702 3173442

[35] M. J. Morris and D. J. Lane , “Tidal Expiratory Flow Patterns in Airflow Obstruction,” Thorax 36, no. 2 (1981): 135–142. 7268679 10.1136/thx.36.2.135 PMC471457

[36] E. Forno , D. J. Weiner , J. Mullen , et al., “Obesity and Airway Dysanapsis in Children With and Without Asthma,” American Journal of Respiratory and Critical Care Medicine 195, no. 3 (2017): 314–323. 27552676 10.1164/rccm.201605-1039OC PMC5328183

[37] E. A. McGinn , E. W. Mandell , B. J. Smith , J. W. Duke , A. Bush , and S. H. Abman , “Dysanapsis as a Determinant of Lung Function in Development and Disease,” American Journal of Respiratory and Critical Care Medicine 208, no. 9 (2023): 956–963. 37677135 10.1164/rccm.202306-1120PP PMC10870865

[38] M. Sanchez‐Solis , E. Forno , E. Morales , and L. Garcia‐Marcos , Group TNS ., “Role of Body Mass Index in Unbalanced (Dysanaptic) Lung Growth of Healthy Infants,” Pediatric Pulmonology 59, no. 11 (2024): 2939–2946. 38967254 10.1002/ppul.27161

[39] K. Wendel , M. E. Rossholt , G. Gunnarsdottir , et al., “Lung Function in Preterm Infants at 3 Months Corrected Age After Neonatal LC‐PUFA Supplementation,” Pediatric Pulmonology 59, no. 2 (2024): 389–398. 37975489 10.1002/ppul.26760

[40] N. R. Bhakta , D. A. Kaminsky , C. Bime , et al., “Addressing Race in Pulmonary Function Testing by Aligning Intent and Evidence With Practice and Perception,” Chest 161, no. 1 (2022): 288–297. 34437887 10.1016/j.chest.2021.08.053 PMC8783030

[41] N. W. Schluger , “The Vanishing Rationale for the Race Adjustment in Pulmonary Function Test Interpretation,” American Journal of Respiratory and Critical Care Medicine 205, no. 6 (2022): 612–614. 35085469 10.1164/rccm.202112-2772ED

[42] L. N. Borrell , J. R. Elhawary , E. Fuentes‐Afflick , et al., “Race and Genetic Ancestry in Medicine — A Time for Reckoning With Racism,” New England Journal of Medicine 384, no. 5 (2021): 474–480. 33406325 10.1056/NEJMms2029562 PMC8979367

[43] R. Kumar , M. A. Seibold , M. C. Aldrich , et al., “Genetic Ancestry in Lung‐Function Predictions,” New England Journal of Medicine 363, no. 4 (2010): 321–330. 20647190 10.1056/NEJMoa0907897 PMC2922981

[44] J. Witonsky , J. R. Elhawary , C. Eng , J. R. Rodríguez‐Santana , L. N. Borrell , and E. G. Burchard , “Genetic Ancestry to Improve Precision of Race/Ethnicity–Based Lung Function Equations in Children,” American Journal of Respiratory and Critical Care Medicine 205, no. 6 (2022): 725–727. 35085059 10.1164/rccm.202109-2088LE PMC12985403

[45] N. R. Bhakta , C. Bime , D. A. Kaminsky , et al., “Race and Ethnicity in Pulmonary Function Test Interpretation: An Official American Thoracic Society Statement,” American Journal of Respiratory and Critical Care Medicine 207, no. 8 (2023): 978–995. 36973004 10.1164/rccm.202302-0310ST PMC10112445

[46] C. Bowerman , N. R. Bhakta , D. Brazzale , et al., “A Race‐Neutral Approach to the Interpretation of Lung Function Measurements,” American Journal of Respiratory and Critical Care Medicine 207, no. 6 (2023): 768–774. 36383197 10.1164/rccm.202205-0963OC

[47] P. H. Quanjer , S. Stanojevic , T. J. Cole , et al., “Multi‐Ethnic Reference Values for Spirometry for the 3‐95‐yr Age Range: The Global Lung Function 2012 Equations,” European Respiratory Journal 40, no. 6 (2012): 1324–1343. 22743675 10.1183/09031936.00080312 PMC3786581

[48] E. Forno , D. J. Weiner , and C. Rosas‐Salazar , “Spirometry Interpretation After Implementation of Race‐Neutral Reference Equations in Children,” JAMA Pediatrics 178, no. 7 (2024): 699–706. 38805209 10.1001/jamapediatrics.2024.1341 PMC11134278

[49] M. Rosenfeld , E. A. Cromwell , M. S. Schechter , et al., “The Impact of Switching to Race‐Neutral Reference Equations on FEV(1) Percent Predicted Among People With Cystic Fibrosis,” Journal of Cystic Fibrosis 23, no. 3 (2024): 443–449. 38556415 10.1016/j.jcf.2024.03.013

[50] Cystic Fibrosis Foundation Patient Registry 2023 Annual Data Report. Bethesda, Maryland; 2024.

[51] D. Albon , A. D. Van Citters , T. Ong , et al., “Telehealth Use in Cystic Fibrosis During COVID‐19: Association With Race, Ethnicity, and Socioeconomic Factors,” supplement, Journal of Cystic Fibrosis 20, no. S3 (2021): 49–54. 34930543 10.1016/j.jcf.2021.09.006 PMC8683127

[52] M. Compton , R. List , E. Starheim , et al., “Home Spirometry Utilisation in Telemedicine Clinic for Cystic Fibrosis Care During COVID‐19 Pandemic: A Quality Improvement Process,” BMJ Open Quality 10, no. 3 (2021). 10.1136/bmjoq-2021-001529 PMC8390143 34433580

[53] J. Medbo , H. Imberg , C. Hansen , C. Krantz , I. de Monestrol , and M. Svedberg , “Telemedicine and Home Spirometry in Cystic Fibrosis: A Prospective Multicenter Study,” Pediatric Pulmonology 59, no. 11 (2024): 2967–2975. 38963304 10.1002/ppul.27166

[54] B. Bouteleux , F. Beaufils , M. Fayon , and S. Bui , “Home‐Spirometry Exacerbation Profiles in Children With Cystic Fibrosis,” Pediatric Pulmonology 59, no. 3 (2024): 552–561. 38014613 10.1002/ppul.26781

[55] A. J. Burbank , J. Laux , J. Brown , M. Sims , S. Ivins , and M. L. Hernandez , “Home Spirometry Monitoring to Identify Loss of Asthma Control in Adolescents,” Pediatric Pulmonology 59, no. 4 (2024): 1117–1119. 38224240 10.1002/ppul.26856 PMC10978234

[56] E. A. Gaillard , C. E. Kuehni , S. Turner , et al., “European Respiratory Society Clinical Practice Guidelines for the Diagnosis of Asthma in Children Aged 5‐16 Years,” The European Respiratory Journal 58, no. 5 (2021): 2004173. 33863747 10.1183/13993003.04173-2020

[57] S. Stanojevic , D. A. Kaminsky , M. R. Miller , et al, “ERS/ATS Technical Standard on Interpretive Strategies for Routine Lung Function Tests,” European Respiratory Journal 60, no. 1 (2022): 2101499. 34949706 10.1183/13993003.01499-2021

[58] N. Beydon and M. Rosenfeld , “Comparison of Bronchodilator Responsiveness in Asthmatic Children Using 2021 or 2005 Ats/Ers Guidelines,” Pediatric Pulmonology 59, no. 1 (2024): 233–235. 37846750 10.1002/ppul.26728

[59] P. H. Quanjer , S. Stanojevic , J. Stocks , et al., “Changes in the FEV(1)/FVC Ratio During Childhood and Adolescence: An Intercontinental Study,” European Respiratory Journal 36, no. 6 (2010): 1391–1399. 20351026 10.1183/09031936.00164109

[60] P. C. Schrader , P. H. Quanjer , B. C. van Zomeren , and M. E. Wise , “Changes in the FEV1‐height Relationship During Pubertal Growth,” Bulletin Europeen De Physiopathologie Respiratoire 20, no. 4 (1984): 381–388. 6332654

[61] A. Ahmed , A. Brown , Y. Pollack , et al., “Relationship Between FEV1/FVC and Age in Children With Asthma,” Pediatric Pulmonology 59, no. 5 (2024): 1402–1409. 38426807 10.1002/ppul.26927

[62] Y. J. Jung , S. W. Ra , S. D. Lee , C. S. Park , and Y. M. Oh , “Clinical Features of Subjects With An Isolated FEV1 Reduction,” International Journal of Tuberculosis and Lung Disease 16, no. 2 (2012): 262–267. 10.5588/ijtld.10.0720 22236930

[63] M. L. Wyatt , A. G. Sokolow , R. F. Brown , et al., “Prevalence, Stability, and Clinical Significance of an Isolated Low FEV1 Spirometry Pattern in Children,” Pediatric Pulmonology 59, no. 6 (2024): 1747–1756. 38558514 10.1002/ppul.26987

[64] M. Abdesslem and H. Ben Saad , “Isolated Low FEV1 in Pediatric Spirometry: A Reflection on Recent Findings,” Pediatric Pulmonology 59, no. 11 (2024): 2726–2728. 38874189 10.1002/ppul.27141

